# Inhibition of actin polymerization decreases osteogeneic differentiation of mesenchymal stem cells through p38 MAPK pathway

**DOI:** 10.1186/1423-0127-20-71

**Published:** 2013-09-26

**Authors:** Himangshu Sonowal, Atul Kumar, Jina Bhattacharyya, Pabitra Kumar Gogoi, Bithiah Grace Jaganathan

**Affiliations:** 1 Stem Cell and Cancer Biology Group, Indian Institute of Technology Guwahati, Guwahati, Assam, India; 2 Department of Hematology, Gauhati Medical College Hospital, Guwahati, Assam, India

**Keywords:** Actin remodeling, MSC differentiation, Integrin CD49E

## Abstract

**Background:**

Mesenchymal Stem Cells (MSC) are important candidates for therapeutic applications due to their ex vivo proliferation and differentiation capacity. MSC differentiation is controlled by both intrinsic and extrinsic factors and actin cytoskeleton plays a major role in the event. In the current study, we tried to understand the initial molecular mechanisms and pathways that regulate the differentiation of MSC into osteocytes or adipocytes.

**Results:**

We observed that actin modification was important during differentiation and differentially regulated during adipogenesis and osteogenesis. Initial disruption of actin polymerization reduced further differentiation of MSC into osteocytes and osteogenic differentiation was accompanied by increase in ERK1/2 and p38 MAPK phosphorylation. However, only p38 MAPK phosphorylation was down regulated upon inhibition of actin polymerization which as accompanied by decreased CD49E expression.

**Conclusion:**

Taken together, our results show that actin modification is a pre-requisite for MSC differentiation into osteocytes and adipocytes and osteogenic differentiation is regulated through p38 MAPK phosphorylation. Thus by modifying their cytoskeleton the differentiation potential of MSC could be controlled which might have important implications for tissue repair and regeneration.

## Background

The multipotential differentiation capacity of mesenchymal stem cells (MSC) makes them important candidates for tissue repair and regeneration of bone [1-3]. Under physiological conditions in the bone marrow, the balance between adipogenesis and osteogenesis of MSC has to be maintained to prevent diseases such as osteoporosis that occurs due to decreased osteogenic differentiation of MSC [1,4,5]. Since adipocytes and osteocytes are a part of the niche cells in the bone marrow, the balance between osteocytes, adipocytes were found to regulate hematopoiesis and tissue homeostasis [6,7]. Thus, a better understanding of the cell intrinsic changes that occur during MSC differentiation is required for cell therapy and tissue repair.

Morphology and cytoskeleton of MSC undergo extensive modifications during differentiation in addition to the gene expression changes [8-11]. Cytoskeletal modification brought about by Rho GTPase has been found to be a major contributor of Mesenchymal Stem Cell (MSC) differentiation and migration [12-14]. During the early stages of differentiation, cues from the microenvironment might affect the differentiation potential and also alter the lineage commitment [15]. The matrix stiffness on which MSC grow has also been reported to direct MSC cell lineage [16]. In addition, substrates with different affinity for the cell surface receptors have been reported to influence MSC differentiation fate. High affinity for the extracellular matrix (ECM) substances by allowing cells to adhere, flatten and spread favored osteogenic differentiation, whereas low affinity for the substrate favored adipogenic differentiation. Cell shape regulated by ECM properties and initial seeding densities has been reported to be important regulators of lineage commitment [17]. Integrins form the actin-linked cell-matrix junctions through which the ECM substances such as fibronectin are linked to actin cytoskeleton [13]. Integrin mediated adhesion to ECM is an essential step that determines the fate of the cells during differentiation [17,18]. Integrin α5 that was upregulated during osteogenic differentiation has been found to be an important regulator of osteogenic differentiation. Silencing of integrin α5 abolished osteogenic differentiation [18].

In this study, we investigated the role of actin cytoskeleton in controlling MSC differentiation and whether lineage specification could be controlled by modifying actin cytoskeleton. We report here for the first time that actin cytoskeleton modification is a very early event during MSC differentiation into adipocytes and osteocytes and might apply to other lineages as well. We found that inhibition of actin polymerization through CYD treatment inhibited osteogenesis by down regulating p38 MAPK but not ERK1/2 MAPK activity.

## Methods

### Chemicals and reagents

Isobutylmethylxanthine, β-glycerophosphate, dexamethasone, ascorbic acid, indomethacin, insulin, paraformaldehyde, human fibronectin, Cytochalasin D (CYD), Phalloidin-tetramethylrhodamine B isothiocyanate (TRITC) and cell culture tested bovine serum albumin (BSA) were purchased from Sigma-Aldrich (Germany). Fluorescence-conjugated monoclonal antibodies for CD13, CD45, CD49d, CD49e, CD73, CD90, CD105, HLA-I, p38MAPK, ERK and NFkB were from BD Biosciences (USA). Reverse transcription reagents were from Applied Biosystems (USA).

### Isolation of MSC from bone marrow

MSC were isolated from patients referred to hematology department of Gauhati Medical College Hospital after ethical consent following local ethical guidelines. The average age of the bone marrow donors was ~28 years. Bone marrow was aspirated from iliac crest and the cells were collected in heparin tubes and after red cell lysis, plated in a tissue culture plate pre-coated with fibronectin (10 ng/cm^2^) in DMEM low glucose medium supplemented with 10% FCS, penicillin and streptomycin. Adherent colonies of spindle shaped cells obtained after 2–3 weeks were sub-cultured and used for further experiments.

### Differentiation and phenotyping

MSC isolated from the BM samples were differentiated into adipogenic and osteogenic lineages as previously described [12]. Media was changed every 2–3 days and adipogenic differentiation was assessed by Oil-red O staining and osteogenic differentiation by alkaline phosphatase staining. The cells were enumerated microscopically to determine the number of differentiated cells.

Bone marrow MSC were phenotyped for the expression of mesenchymal cell surface markers by flow cytometry. The cells were trypsinized and stained with fluorescently conjugated monoclonal antibodies against CD13, CD29, CD45, CD49a, CD49b, CD49e, CD73, CD90, CD104, CD105 and HLA-I. The cells were incubated on ice for 30 minutes, washed and analysed by FACS calibur (BD Pharmingen). Propidium iodide was used for live/dead discrimination.

### Phospho staining for flow cytometry

Cells were trypsinized and fixed immediately with 4% formaldehyde and permeabilised with 100% methanol. The cells were stained with fluorescent conjugated antibodies that specifically bind to the phosphorylated form of proteins for 1 hour at room temperature and analysed by flow cytometry.

### Actin staining

Cells grown on fibronectin coated cover slips or plates were fixed with paraformaldehyde (4%), permeabilised with Triton X-100 (0.1%) and stained with TRITC conjugated phalloidin overnight at 4°C. After washing with PBS, the cells were mounted and documented using Nikon CCD camera.

### Real-time quantitative PCR

Quantitative real-time PCR was performed to quantify the transcript level of different genes. RNA was isolated using Trizol reagent and reverse transcribed into cDNA using MultiScribe reverse transcriptase (Applied Biosystems, USA) and real-time PCR was performed using SYBR Green reagents (Applied Biosystems, USA). The primers used were: GAPDH forward 5′-GGGAAGGTGAAGGTCGGAGT-3′, GAPDH reverse 5′-GGGTCATTGATGGCAACAATA-3′, ACTIN forward 5′- GCACAGAGCCTCGCCTTT-3′, ACTIN reverse 5′- CGCCCACATAGGAATCCTTC-3′, ADIPONECTIN forward 5′-CCATCTCCTCCTCACTTCCA-3′, ADIPONECTIN reverse 5′-GTGCCATCTCTGCCATCAC-3′, PPARg forward 5′-GACCACTCCCACTCCTTTGA-3′, PPARg reverse 5′-CGACATTCAATTGCCATGAG-3′, OSTEOCALCIN forward 5′-GTGCAGAGTCCAGCAAAGGT-3′ and OSTEOCALCIN reverse 5′-TCAGCCAACTCGTCACAGTC-3′.

### Inhibition experiments

Inhibition of actin polymerization was performed by addition of CYD (Sigma) for different time points at various concentrations. For recovery after CYD treatment, the cells were washed twice with PBS, normal growth media or induction media was added for the indicated time periods.

### Scanning Electron Microscopy (SEM)

Cells were cultured on fibronectin coated coverslips, fixed with 2.5% gluteraldehyde and dehydrated with graded series of ethanol (30%, 50%, 70%, 90% and 100%). The cells were then gold coated with a sputter coater and viewed under Scanning Electron Microscope (Leo 1430vp, Germany).

### Statistical analysis

Statistical analysis was performed using SPSS software and values of p < 0.05 were considered statistically significant.

## Results

**Figure 1:**
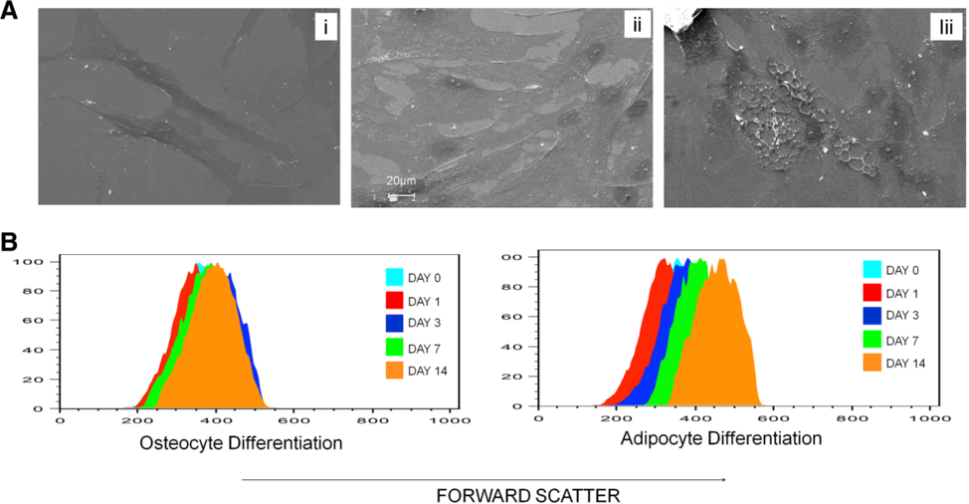
**Morphology and cell size during MSC differentiation. (A)** Scanning electron microscope images of MSC differentiated into osteocytes or adipocytes. The cells were left uninduced (i) or induced into either osteocytes (ii) or adipocytes (iii) in their respective induction media for 14 days. Osteocytes attained an angular shape whereas the adipocytes become globular in shape with oil droplet. Representative image is shown. **(B)** Flow cytometric analysis of cell size by forward scatter measurement in MSC differentiated into either osteocytes or adipocytes. The cells were analysed after day 0, 1, 3, 7, 14 days of induction into osteocytes or adipocytes. Representative histograms are shown.

To understand the molecular events preceding and driving the differentiation of MSC into various lineages, we studied the role of actin cytoskeleton during differentiation of MSC into osteocytes and adipocytes. For this, human MSC were cultured in adipogenic or osteogenic induction medium and their change in morphology and cytoskeleton was monitored during early and late stages of differentiation. SEM images of differentiated cells clearly revealed that differentiated cells have an altered morphology. After 14 days of culture in differentiation media, the cells attained a globular shape during adipogenesis and the cells became angular with increased cell extensions during osteogenesis whereas the undifferentiated cells were spindle shaped (Figure 1A). We found that during adipogenic differentiation, the cells increased in their size gradually until day 14 whereas during osteogenic differentiation, the cell size remained relatively unaltered (Figure 1B).

**Figure 2:**
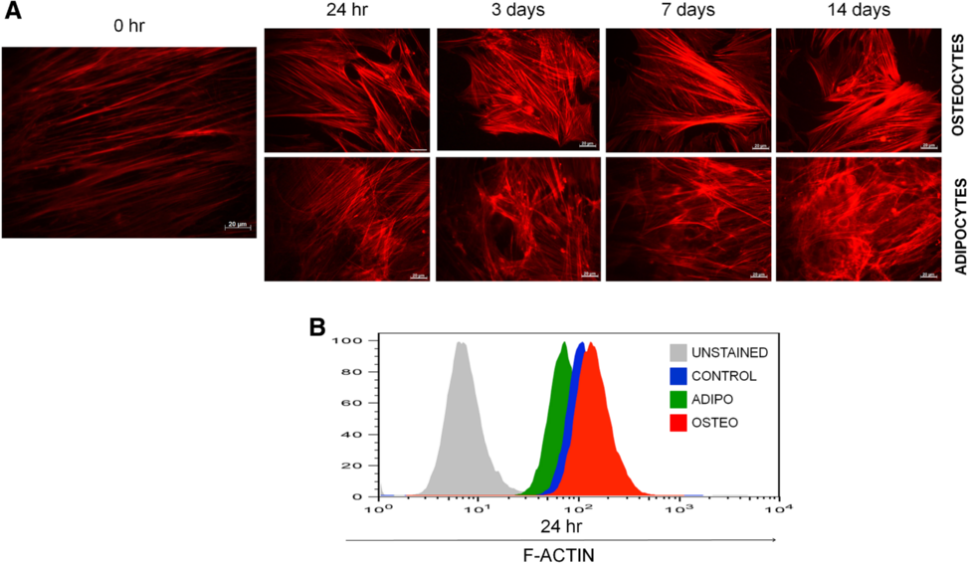
**Actin cytoskeleton rearrangement during MSC differentiation. (A)** MSC were grown in media containing osteogenic or adipogenic inducers for 24 hours, 3 days, 7 days and 14 days or left uninduced (0 hr) and F-actin was visualised by staining with phalloidin**-TRITC.** Photomicrographs are representative images from 3 independent experiments. **(B)** Flow cytometric analysis of uninduced (CONTROL) or MSC induced with osteogenic or adipogenic media for 24 hour and stained with phalloidin-TRITC. X-axis represents the F-actin fluorescence intensity, representative graphs are shown. Unstained cells were used to obtain the auto fluorescence levels during flow cytometry.

To determine the initial cellular changes that occur during MSC differentiation, we analysed the status of actin cytoskeleton by staining the cells with TRITC conjugated phallodin at various stages of differentiation. Undifferentiated MSC *in vitro* showed parallel actin filaments traversing the entire length of the spindle shaped cells as seen in Figure 2A. In undifferentiated MSC, the actin cytoskeleton arrangement remained unaltered during various passages, however, within 24 hours of induction into adipocytes or osteocytes, the cells underwent significant actin cytoskeleton modification (Figure 2A) which was accompanied by increase in formation of oil droplets in the adipo-induced cells or alkaline phosphatase activity in osteo induced cells. Actin cytoskeleton remodeling continued until 14–21 days where osteogenic induction resulted in the formation of peri-nuclear actin bundles framing the angular cell body showing abundant stress fibres and increased actin polymerization (Figure 2A). During adipogenic differentiation, the cells showed discontinuous actin filaments forming a network like structure. When the cells started accumulating oil-droplets, actin filaments formed a disrupted network around the oil-droplets (Figure 2A). The changes in actin modification were very early during differentiation where the filamentous actin (F-actin) concentration increased within 24 hours during osteogenesis but decreases during adipogenesis (Figure 2B). Thus the change in morphology, cell shape, size and actin remodeling were important cellular events that defined MSC differentiation into adipocytes or osteocytes.

**Figure 3:**
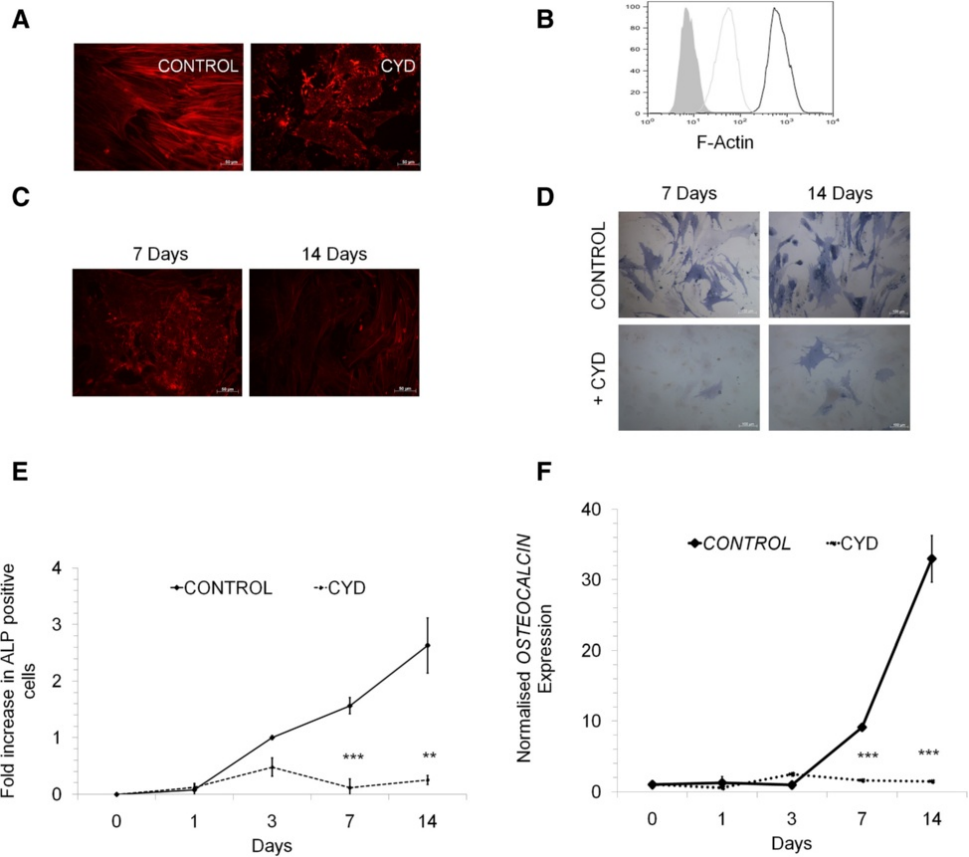
**Effect of CYD treatment on osteogenic differentiation. (A)** MSC were left untreated (CONTROL) or treated with CYD (100 ng/ml) for 24 hours in the normal growth media and stained with phalloidin-TRITC showing less F-actin in CYD treated cells. **(B)** Flow cytometric analysis of MSC untreated (grey line) and treated with CYD (100 ng/ml, black line) for 24 hours stained with phalloidin-TRITC and x-axis shows the TRITC fluorescence. Grey filled is unstained control. **(C)** MSC were induced to undergo osteogenesis without (CONTROL) or with CYD (+CYD) for 7 or 14 days and F-actin visualised by staining with phalloidin-TRITC. **(D)** MSC were induced to undergo osteogenesis without (CONTROL) or with CYD (+CYD) for the indicated time period showing the fold increase in ALP positive cells. Blue colour represents alkaline phosphatase stained cells. ALP positive cells **(E)** or Osteocalcin expression **(F)** in MSC induced to undergo osteogenic differentiation without (CONTROL) or with CYD (CYD) for indicated time periods. X-axis **(E**,**F)** represents the number of days in differentiation media and y-axis indicates the fold increase in ALP positive cells compared to day 1 (CONTROL, E) or normalised OSTEOCALCIN expression **(F)**. Representative photomicrographs or flow cytometric histogram is shown. ALP stands for alkaline phosphatase. Values are mean ± SD, n = 3-4. ** P < 0.005, *** p < 0.0005.

Given the significant differential changes in the actin cytoskeleton during osteogenic or adipogenic differentiation of MSC as early as 24–48 hours of induction, we sought to find out if actin remodelling was a pre-requisite for MSC differentiation and if differentiation could be controlled by actin cytoskeleton modification. Although the actin remodelling initiated within 24 hours of induction of differentiation (Figure 2A), the changes in gene expression was very minimal. To understand the role of actin remodelling in driving or inhibiting the differentiation of MSC into either osteocytes or adipocytes, the cells were treated for different time periods with CYD, in the presence or absence of induction media. Inhibition of actin polymerisation was evident within 24 hours of treatment of MSC with CYD and effective concentration was found to be 100–1000 ng/ml without compromising the cell viability (Figure 3A). Flow cytometric analysis showed decreased fluorescence in cells treated with CYD compared to control cells when stained for F-actin (Figure 3B). This effect of CYD on actin polymerisation could be reversed when the inhibitor was removed and cells were allowed to recover in the respective induction media or normal media (Data not shown).

**Figure 4:**
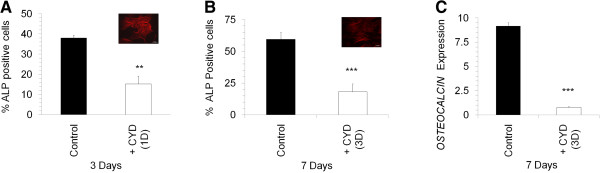
**Effect of CYD treatment on osteogenic differentiation.** Percentage of ALP positive cells **(A**,**B)** or *OSTEOCALCIN* (C) expression in MSC induced to undergo osteogeneis without (CONTROL) or with CYD (+CYD) treatment. CYD treatment was given for initial one day (1D) during 3 days of osteogenic induction **(A)** or for initial 3 days (3D) during 7 days osteogenesis induction **(B**,**C)**. F-actin staining with phalloidin-TRITC of CYD treated MSC in each condition is shown in the inset. Values are mean ± SD, n = 3-4. ** P < 0.005, *** p < 0.0005, ALP stands for alkaline phosphatase.

Interestingly, when MSC were treated with CYD for 7 days in the presence of osteogenic induction media, there was a significant reduction in osteocytes as evidenced by decrease in alkaline phosphatase positive cells (Figure 3D-F). When CYD treatment period was extended up to 14 days in osteogenic induction media, there was a 10-fold reduction in the osteogenic differentiation showing little or no actin filaments in the treated samples (Figure 3C-E). Consistent with the decreased alkaline phosphatase activity, there was a significant decrease in *OSTEOCALCIN* levels when the cells were treated with CYD for different durations (Figure 3F).

We found that 24 hours of CYD treatment was sufficient to reduce osteoblast differentiation by 50% even though the cells were allowed to recover for 48 hours without CYD in the osteogenic induction media (Figure 4A). However, this recovery period of 48 hours in the induction media was sufficient to allow the remodelling of actin where polymerised actin (F-actin) was seen in the differentiating cells. Furthermore, when the cells were treated with CYD for 3 days and allowed to recover for 4 days in the induction media, there was 3-fold decrease in the osteogenic differentiation potential where actin cytoskeleton rearrangement appeared normal (Figure 4B, C).

**Figure 5:**
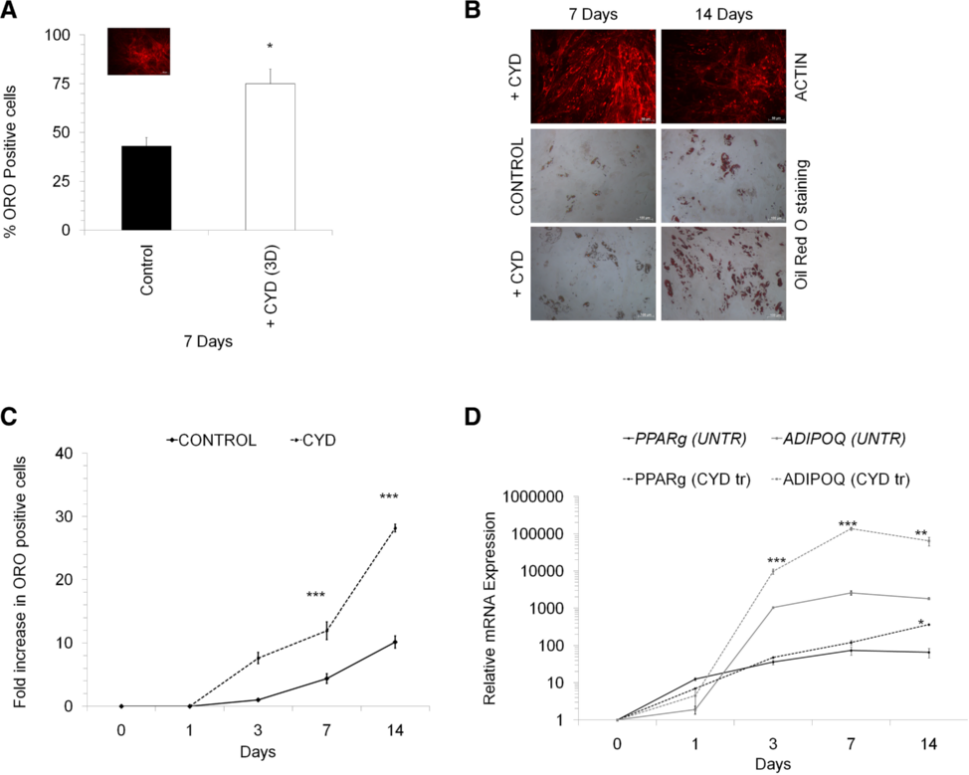
**Effect of CYD treatment on adipogenic differentiation. (A)** Percentage of MSC differentiated into adipocytes for 7 days without (CONTROL) or with CYD treatment (+CYD) was determined by ORO staining where CYD treatment was done for the initial 3 days (3D) during the 7 days induction period. F-actin staining with phalloidin-TRITC for CYD treated MSC is shown in the inset. **(B)** F-actin and ORO staining in MSC induced to differentiate into adipocytes without (CONTROL) or with CYD treatment (+CYD) for 7 or 14 days. Representative photomicrographs are shown. **(C)** Fold increase in ORO positive cells in MSC were induced to undergo adipogenesis without (CONTROL) or with CYD (+CYD) for the indicated time periods: **(D)** ADIPOQ and PPARG mRNA levels in untreated (CONTROL) or CYD treated (+CYD) MSC induced to undergo adipogenesis for the indicated time periods. Values are mean ± SD, n = 3-4. * p < 0.05, ** P < 0.005, *** p < 0.0005, ORO stands for Oil red O.

In contrast, when the cells were treated with CYD during adipogenic differentiation there was a significant increase in the oil-Red O positive adipocytes. Three days of initial CYD treatment during 7 days of adipogenic induction was sufficient to increase adipogenic differentiation by ~30% (Figure 5A). During the recovery period, the actin cytoskeleton reverted back to its cross linked form as seen in normal adipocytes. To understand further the effect of CYD treatment on adipogenic differentiation, MSC were treated with cytochalsin D for 7 days, that is, throughout the adipogenic induction period. Surprisingly we found that there was a 3-fold increase in the adipogenic differentiation (oil-Red O positive cells) compared to untreated controls. To further confirm the possibility that inhibition of cytoskeleton increases the adipogenic differentiation capacity of MSC, the treatment with CYD was extended to 14 days in the induction media. Consistent with the 7 days result, there was a 2.8 fold increase in the adipogenic differentiation of MSC when they were treated with CYD for 14 days during induction compared with the cells cultured with the normal induction media (Figure 5B, C). Notably, the cells treated with CYD for 7 days or 14 days without the recovery period lacked actin polymerisation when stained with phalloidin TRITC (Figure 5B). To confirm an increase in adipogenic differentiation during CYD treatment, we quantified the mRNA levels of adiponectin (*ADIPOQ*) and peroxisome proliferator-activated receptor gamma (*PPARG*) in adipo-induced cells. Consistent with the increased oil-red O positive cells, there was a subsequent increase in the expression levels of *ADIPOQ* and *PPARG* in CYD treated cells (Figure 5D).

In order to determine whether inhibition of actin polymerization prior to induction of differentiation could affect the differentiation potential of MSC, we pre-treated MSC with CYD for 3 days and allowed the cells to differentiate into osteocytes and adipocytes in the absence of CYD. There was an increase in adipogenic differentiation potential and a significant decrease in the osteogenic differentiation potential was observed (Additional file 1: Figure S1 A-C). In addition, CYD pre-treatment in the absence of induction factors was sufficient to decrease *OSTEOCALCIN* expression but induce *PPARG* expression in MSC (Additional file 1: Figure S1 D, E). This confirms the earlier observation that cytoskeletal modification was an early event during MSC differentiation.

**Figure 6:**
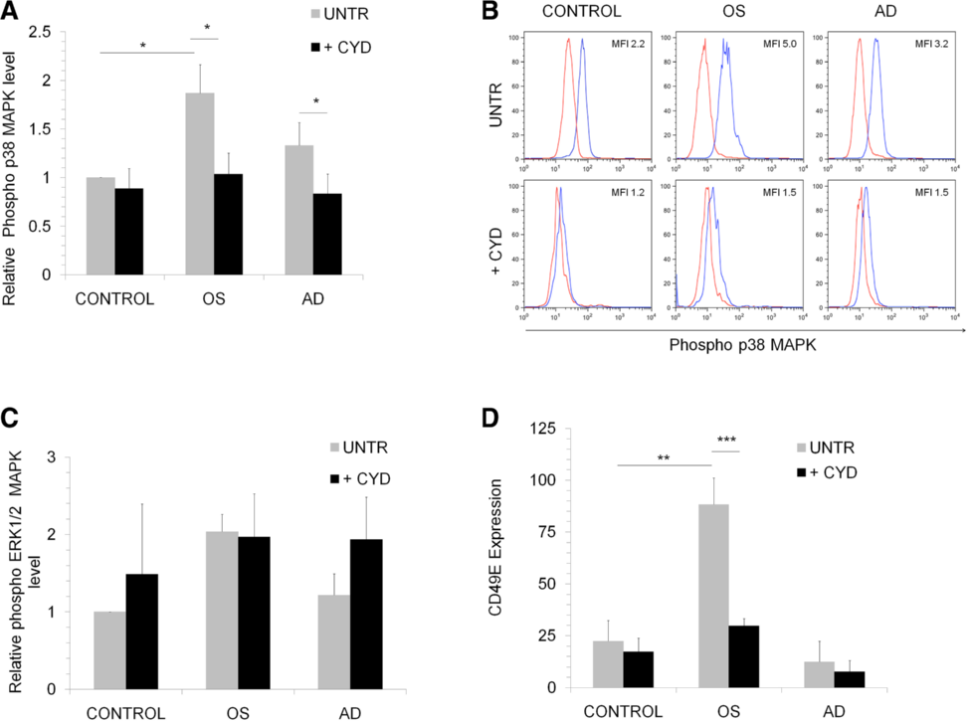
**p38MAPK and CD49E expression changes during MSC differentiation.** MSC were differentiated into osteocytes (OS) or adipocytes (AD) in the absence (UNTR) or presence of CYD (+CYD) for 3 days and the phosphorylated levels of p38 MAPK **(A)** and ERK1/2 MAPK **(C)** was determined by flow cytometry. Y-axis in figure **A** and **C** shows the relative phosphorylated levels of p38 MAPK or ERK1/2 MAPK compared to undifferentiated untreated control cells (CONTROL). **B**. Representative flow cytometric histrograms of MSC differentiated into osteocytes (OS) or adipocytes (AD) which were left untreated (UNTR) or treated with CYD (+CYD) stained for phosphorylated form of p38MAPK. Red line is the isotype control and blue line is the sample. **D**. Flow cytometric analysis of CD49E surface expression in MSC differentiated into osteocytes (OS) or adipocytes (AD) which were left untreated (UNTR) or treated with CYD (+CYD) for 14 days. Values are mean ± SD, n = 3, *p < 0.05, **p < 0.005, *** p < 0.0005.

To understand the molecular pathways affected by actin modification we studied the activation levels of NFκB, p38 and ERK1/2 MAPKs during MSC differentiation into adipocytes or osteocytes. We found that phosphorylated levels of p38 and ERK1/2 MAPKs increased during osteogenesis (Figure 6A-C) but no significant difference was seen in NFκB phosphorylation. On treatment with CYD, there was a significant decrease in the phosphorylated levels of p38MAPK not ERK1/2 MAPK during both osteogenesis and adipogenesis. Hence, we can conclude that even though phopshorylated levels of both p38 and ERK1/2 MAPK increased during osteogenesis, it is through p38MAPK pathway in MSC, CYD downregulates osteogenic differentiation (Figure 6A-C).

Actin is linked to the external micro environment through integrins and reports suggest that integrins mediate cytoskeleton organization, gene expression and differentiation [19,20] and so we sought to find out the changes in integrin expression during osteogenesis and adipogenesis. There was a four-fold increase in the fibronectin receptor CD49E when the cells differentiated into osteoblasts whereas no significant difference was observed in adipo differentiated cells. We found a 50% reduction in CD49E expression in osteoblasts when the cells were treated with CYD during differentiation (Figure 6D).

Taken together, our results strongly suggest that cytoskeletal changes are very important for MSC differentiation into adipocytes and osteocytes and it is a very early cellular event which preceeds the gene expression changes. Actin modification seems to regulate osteogenic differentiation through p38 MAPK pathway.

## Discussion

In the current study, we report that changes in cell shape and actin cytoskeleton remodeling were important events during MSC differentiation into adipocytes and osteocytes. Cytoskeleton modification was an early event during differentiation and it occurred as early as 24 hours after the addition of respective induction media. The cytoskeleton was differentially modified during osteogenic and adipogenic differentiation where there was more actin polymerization and the cells acquired more stress fibres and actin bundles were clearly visible during osteogenic differentiation. However during adipogenesis, there was a reduction in actin polymerization where actin filaments occurred as a broken network like structure. Although the cell size increased during adipogenic differentiation, more F-actin was formed during osteogenic differentiation. These differences suggest that these changes might be due to the different mechanical strength required for osteocytes and adipocytes [21]. Increased polymerization and stress fibres might render osteocytes which form the bones in the body with more mechanical strength required to withstand the physical stress [9]. Although RHO GTPases have been found to be involved in regulating the differentiation of MSC [14], our results suggest that cytoskeleton modification seems to be the early event directing the differentiation of these cells.

Engler et al. reported that matrix elasticity determines the lineage commitment in MSC [16], but our experiments clearly show that under uniform matrix elasticity, the cytoskeletal organization directs the lineage commitment. Although changes in the cytoskeleton during osteogenesis has been reported [8,9], we show here that cytoskeletal modification is not an effect of differentiation but a contributing factor for differentiation. Gene expression studies also confirm the observation that cytoskeleton modification through CYD treatment was sufficient to modify MSC differentiation by increasing *PPARG* levels and decreasing *OSTEOCALCIN* levels. Although the cells could differentiate into osteocytes after the removal of CYD, we observed a significant decrease in the differentiation potential which could be attributed to the decrease in *OSTEOCALCIN* levels during CYD treatment. Previous studies have shown that beta-actin attaches itself to the surface of fat droplets indicating a possible role for beta-actin in lipid metabolism [22]. In addition, steroid responding cells which include adipocytes seem to maintain a higher level of monomeric actin which facilitates cholesterol transport [23,24]. This might be the reason for increased adipogenesis and increase in *PPARG* levels seen in our study when actin polymerization into F-actin was inhibited by CYD treatment resulting in higher amounts of G-actin in the form of beta-actin in the cells.

We also found that osteogenic differentiation caused an up regulation of CD49E as reported by others [18] and CYD treatment resulted in reduced osteogenic potential of cells which in turn might have caused the decrease in CD49E expression. From this experiment we conclude that cytoskeletal changes precede gene expression and integrity of actin cytoskeleton was required for osteogenic differentiation as reported also in other cell types [20]. An interesting finding in our study is that decreased actin polymerization facilitated adipogenesis in contrast to its inhibiting effects on osteogenesis.

Yang et al. suggested that actin binding could regulate p38 MAPK activity [25] and several studies reported the importance of p38 MAPK in regulating osteogenic differentiation. P38 MAPK activity positively regulated BMP-2, BMP-9 induced osteogenic differentiation [26-28] whereas it was found not to be important for mechanical strain induced osteogenesis [29]. Although we did not find any significant decrease in osteogenesis on addition of p38 MAPK inhibitor SB208530 (data not shown), there was an increased phosphorylation of p38 MAPK which was effectively down regulated by actin polymerization inhibition. However, the role of actin in regulating p38 MAPK during osteogenesis requires further study.

## Conclusion

Taken together, our results suggest that differential actin remodeling occurs during MSC differentiation which precedes the gene expression changes. This actin modification regulates p38 MAPK phosphorylation which can be modified by CYD treatment. The combined effect of actin polymerization and p38 phosphorylation regulates osteogenic differentiation.

## Competing interests

The authors declare no competing financial interests.

## Authors’ contributions

HS performed the experiments, AK performed the phosphorylation studies, analysed the data and wrote the manuscript; JB and PKG provided vital samples and data; BGJ conceived, designed the experiments, analysed the data and wrote the manuscript. All authors read and approved the final manuscript.

## Supplementary Material

Additional file 1: Figure S1 MSC were pre-treated with CYD (+CYD (3D)) for 3 days and allowed to differentiate into osteocytes (A, B) or adipocytes (C) for 14 days in the respective induction media without CYD. Osteogenic and adipogenic differentiation was determined by staining for alkaline phosphatase (ALP) and oil-red O (ORO) respectively. Values are mean ± SD, n = 3. Representative microphotographs are shown, the bar represents 200μm. Real-time PCR analysis of *OSTEOCALCIN* (D) and *PPARG* (E) expression levels in MSC treated without (UNTR) or with CYD (+CYD (7D)) for 7 days. Values are mean ± SD, n = 3. * p < 0.05, ** P < 0.005. Click here for file
