# Supplementary figures and images for: Inhibition of actin polymerization decreases osteogeneic differentiation of mesenchymal stem cells through p38 MAPK pathway
Source: J Biomed Sci. 2013 Sep 26;20(1):71. doi: 10.1186/1423-0127-20-71 (PMC3849435; doi:10.1186/1423-0127-20-71)

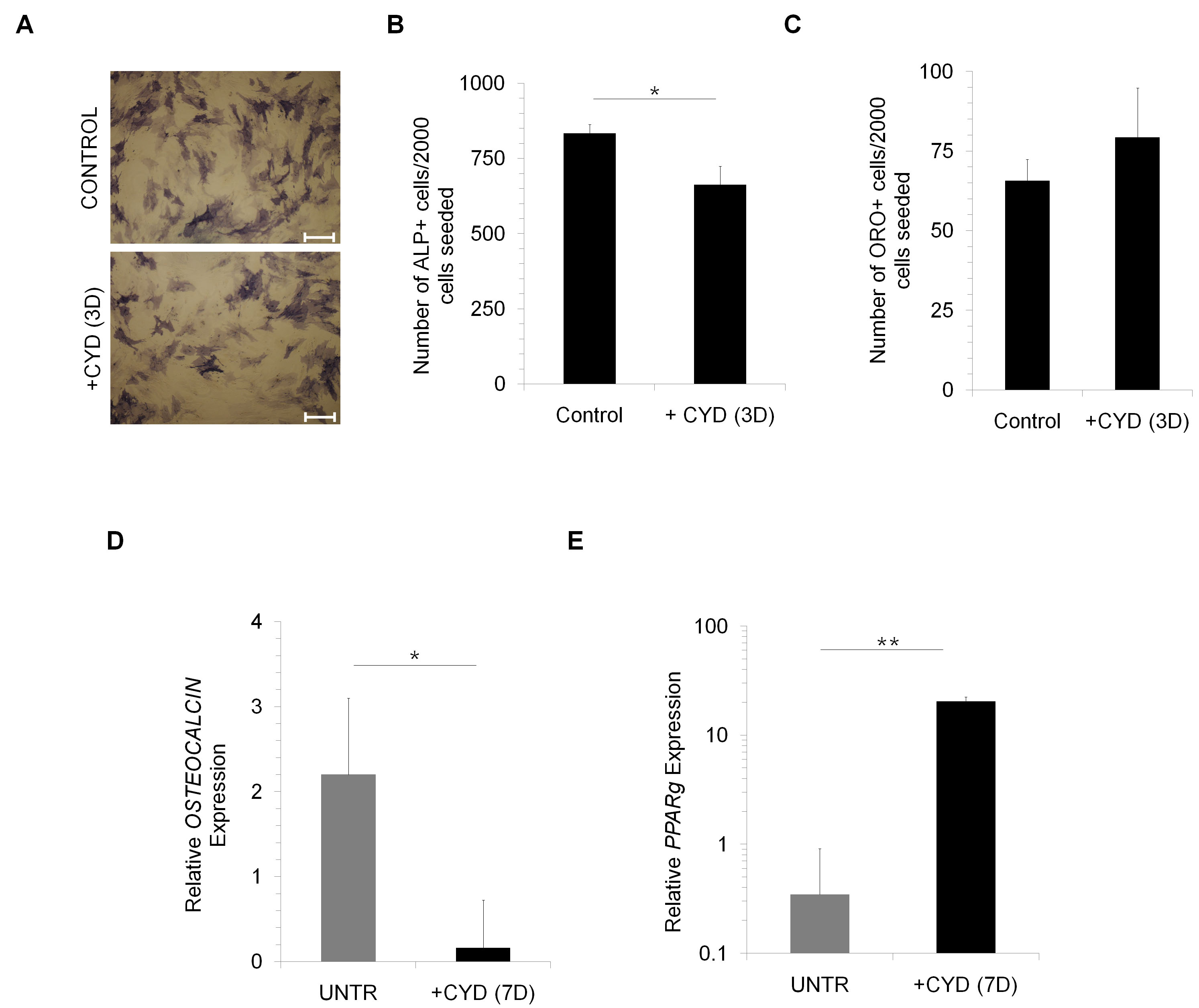

Supplement: Additional file 1: Figure S1 — MSC were pre-treated with CYD (+CYD (3D)) for 3 days and allowed to differentiate into osteocytes (A, B) or adipocytes (C) for 14 days in the respective induction media without CYD. Osteogenic and adipogenic differentiation was determined by staining for alkaline phosphatase (ALP) and oil-red O (ORO) respectively. Values are mean ± SD, n = 3. Representative microphotographs are shown, the bar represents 200μm. Real-time PCR analysis of OSTEOCALCIN (D) and PPARG (E) expression levels in MSC treated without (UNTR) or with CYD (+CYD (7D)) for 7 days. Values are mean ± SD, n = 3. * p < 0.05, ** P < 0.005. [file 1423-0127-20-71-S1.jpeg]
